# Patient-reported outcomes of treatment and adverse effects following acute lymphoblastic leukemia: a low- and middle-income country cross-sectional study

**DOI:** 10.1016/j.htct.2024.05.006

**Published:** 2024-07-29

**Authors:** Eduardo Cerello Chapchap, Nina Melo, Denise Martins, Maria Lucia Lee, Nelson Hamerschlak

**Affiliations:** a Hospital Israelita Albert Einstein, Dayan-Daycoval Hematology and Oncology Center, Brazil; b Associação Brasileira de Linfoma e Leucemia (ABRALE), Brazil; c Hospital Beneficencia Portuguesa de Sao Paulo, Department of Hematology, Brazil

**Keywords:** Patient reported outcomes, Acute lymphoblastic leukemia, Treatment, Adverse effects

## Abstract

**Introduction:**

The scenario of adult patients with acute lymphoblastic leukemia treated in Brazil has not been well described yet.

**Methods:**

Four hundred patients diagnosed with acute lymphoblastic leukemia from 1981 to 2019, registered in the Brazilian lymphoma and leukemia association (ABRALE) or their caregivers were interviewed by telephone to evaluate patient-reported perceptions of diagnosis, treatment and adverse effects.

**Results:**

Overall, 203 were male with a mean age of 15.7 years and median follow-up of 6.2 years. Main presenting symptoms were fever (39 %), bleeding/ecchymosis (38 %), intense fatigue (30 %), and musculoskeletal pain (28 %). The proportion of patients diagnosed within one week of symptoms onset differed between public (17.9 %) and private healthcare (31.1 %; p-value = 0.019). Additionally, diagnostic difficulties were higher in public care: 35 % versus 22.6 % (p-value = 0.034). Only 36 patients were able to report their treatment protocols; from a list of eight reported protocols, the most common were the Brazilian Childhood Cooperative Group for Treatment of Acute Lymphoblastic Leukemia in Children (GBTLI - 10/27.8 %) and Berlin-Frankfurt-Münster (BFM - 8/22.2 %). Seventy patients (17.5 %) required treatment modification, 37.1 % due to severe adverse effects; 21.7 % received short treatment duration (≤6 months) and 16 % proceeded to allogeneic hematopoietic stem cell transplantation with 17/64 (27 %) reporting difficulties in this step, characterized as >3 months delay. Indication for transplantation was related to minimal residual disease and cranial radiotherapy; 41.7 % reported treatment-related adverse effects (range: 1–6), in particular: mood disorders (26.3 %), neurologic deficit (13.8 %), cognitive/memory impairment (12 %), and lung disease (15 %). Risk factors for adverse effects were age, indication of transplantation and living in a large city. Treatment disparities such as diagnostic and transplantation delays remain challenges in these patients.

**Conclusions:**

Urgent interventions are needed to optimize healthcare and reduce adverse effects, especially in adolescent and young adult patients.

## Introduction

The scenario of adult patients with acute lymphoblastic leukemia (ALL) treated in Brazil has not been well described yet.^1^^,^^2^ In the pediatric setting, national treatment protocols are under development and being improved since the 1980s. In particular the Brazilian Childhood Cooperative Group for Treatment of Acute Lymphoblastic Leukemia in Children (GBTLI) protocol has been standardized nationwide.^3^ In contrast, there are no national standardized protocol and registry database for adult ALL patients. Indeed only a few single-center studies have been published.^2^, ^3^, ^4^, ^5^

Brazil is a very large country, where many unsolved socioeconomic issues and disparities result in a very heterogeneous distribution of healthcare facilities between treating centers. Different cultural and educational aspects also add to this heterogeneity thereby affecting the relationship between patients and healthcare professionals, depending on the region where they live.^1^^,^^2^ As ALL is a rare disease in adults, it is more difficult to increase our knowledge in non-pediatric patients.^1^^,^^2^^,^^4^

Attempts have been made to standardize the treatment of ALL in adults to try to resolve this issue, including the BRALL 2014 protocol initiative and a multicentric study investigating the effectiveness of recombinant l-asparaginase.^5^ However, disparities in the management of ALL persist, probably due to large differences in the availability of healthcare facilities across regions; this also prejudices compliance to diagnostic and treatment protocols. Taken together, these issues make it difficult to develop national guidelines and registry databases, and to investigate how patients move inside the country seeking medical attention or referrals to ALL excellence centers.

Despite of all the advances in the treatment of ALL, literature addressing patients’ perceptions, from diagnosis to long-term follow-up, is lacking.^6^ Furthermore, patient-reported outcomes could be very valuable to elucidate which issues are actually impacting their healthcare, their lives and wellbeing.^6^, ^7^, ^8^ Patient feedback on problems, difficulties and adverse events faced during ALL treatment would complement the physicians’ perspective, offering essential information for healthcare providers and allowing improvements in protocols.^6^, ^7^, ^8^ By investigating patient-reported outcomes and increasing the knowledge on the scenario of Brazilian ALL patients, it will be possible to further improve the standard of care and propose strategies to overcome barriers and reduce adverse effects.

### Objective

The objective of this study was to investigate the perceptions of Brazilian ALL patients about diagnosis, treatment and adverse events and to analyze associations between these variables and patient demographics, disease status and risk of adverse events.

## Methods

### *Design, ethics and setting*

This cross-sectional study included all patients with ALL registered in the Brazilian Lymphoma and Leukemia Association (ABRALE) until 2019. ABRALE is a non-governmental civil society organization (NGO) that supports patients with leukemia and lymphoma and their families by offering educational programs, publications and scientific/interactive events in Brazil. Patients can contact ABRALE and be registered in the database in different ways. ABRALE representatives in treating centers approach some patients while others contact ABRALE to get information, guidance or help on healthcare issues.

This study protocol was approved by the Teaching and Research Ethics Committee of the Albert Einstein Institute. This study was supported and sponsored by ABRALE and AMIGOH (Friends from Oncology and Hematology). Informed consent was obtained and recorded by telephone from all participants during interviews. Data are treated with confidentiality and only anonymous aggregated data are reported here.

### *Participants*

**Figure 1:**
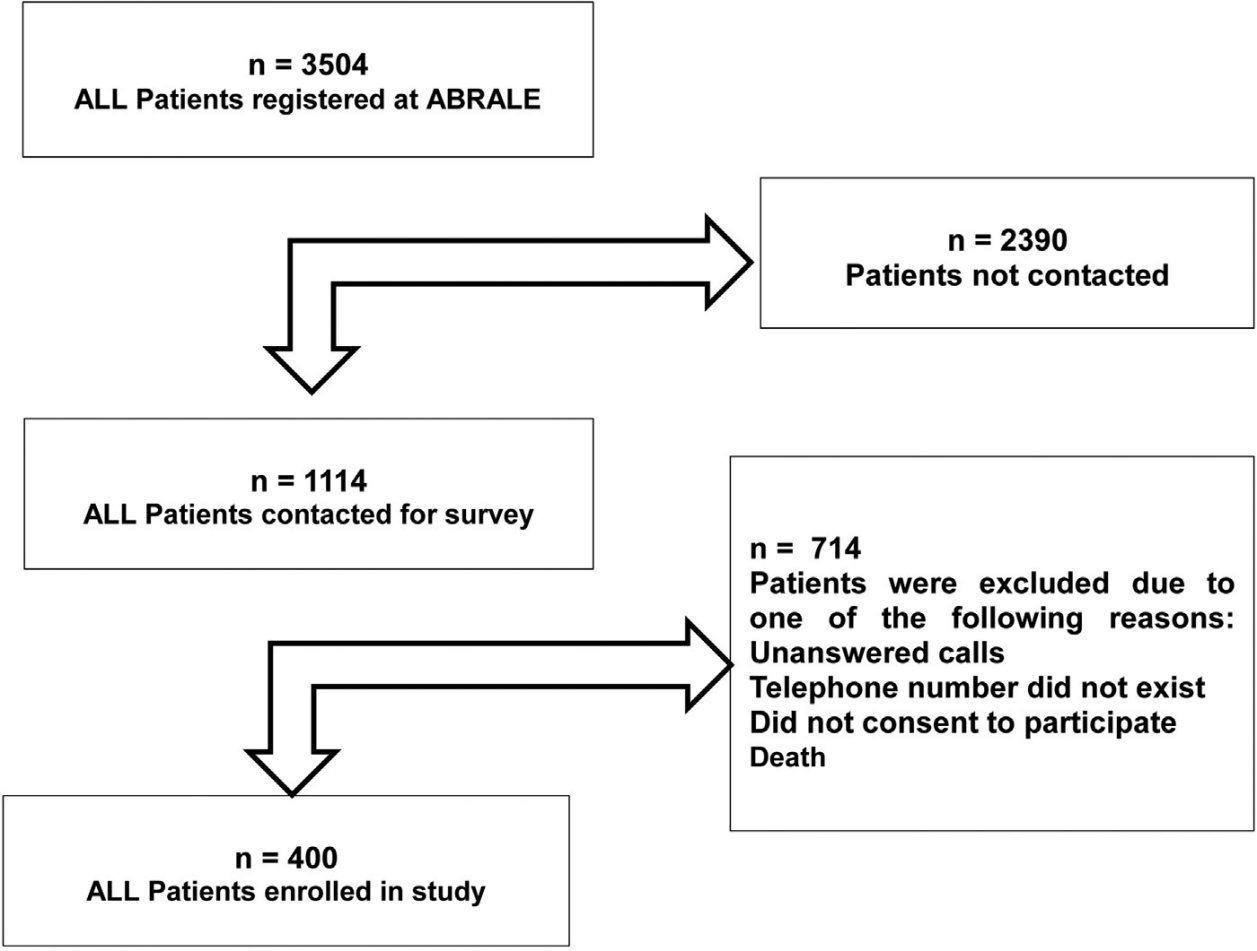
Patients eligible and enrolled in this study.

For this study, all patients diagnosed with ALL and registered with ABRALE were recruited for this study in December 2019. At least three attempts were made to contact patients. All were interviewed by telephone about their experience of the care they were receiving for ALL. Alternatively, caregivers were interviewed, which was generally the case for under 17-year-old patients. Patients were excluded if contact information on file was out-of-date (out-of-reach) or if they did not consent to participate in the study. The flowchart summarizes how many patients were eligible and enrolled in this study. Enrolled patients were treated from 1981 until January 2019 and registered in ABRALE database (Figure 1).

The majority of patients were from nine large Brazilian cities (Rio de Janeiro, Curitiba, Goiânia, Recife, Porto Alegre, Belo Horizonte, São Paulo, Campinas and Salvador) or from smaller cities in the states of Amazonas, Ceará, Espírito Santo, Maranhão, Mato Grosso, Mato Grosso do Sul, Pará, Paraíba, Pernambuco, Santa Catarina, Rondônia and Tocantins.

### *Interviews and questionnaire*

A specific questionnaire was designed for this study to collect data related to patient demographics, the healthcare system, disease status, treatment management and related difficulties, satisfaction and adverse events. Some of the questions were adapted from the quality-of-life instrument of the European Organization for Research and Treatment of Cancer (EORTC QLQ-C30).^9^ Interviews were conducted in Portuguese (Table 1).

**Table 1:** Questionnaire.

**1. Name**
**2. Gender**
**3. Birth of date**
**3.1. Interview date**
**3.2. Age at interview**
**4. Where (state and city) do you live?**
**6. Are you the patient or the patient's caregiver?**
**6.1. What is your relationship with the patient?**
Mother/father
Uncle/aunt
Brother/sister
Husband/wife
Friend
Grandfather/grandmother
Son/daughter
Grandchild/granddaughter
Caregiver
Sister/brother-in-law
**7. Did you have any symptoms that led you to seek medical attention before diagnosing the leukemia?**
**7.1. Which were those symptoms?**
Pallor
Excessive tiredness/fatigue
Sleepiness
Hematomas/bruises/ecchymosis
Petechiae (small red dots on your skin)
Bleeding/hemorrhage
Recurrent infections
Swollen lymph nodes
Swollen spleen
Headache
Vomits
Bone pain/joint pain
Fever
Leg or arm pain
Loss of appetite
Anemia detected by lab test
Throat infections/swollen tonsils
Weight loss
Cough
Facial palsy
Leg palsy/movement loss
Distended abdominal
Weakness
Dizziness
Syncope/fainting
Flu-like symptoms
Dyspnea/shortness of breath
Otitis
Diarrhea/constipation
Chest pain
Stomach pain
Abdominal pain
Heart rhythm abnormalities/arrhythmia
Body nodules
Oral lesions
Nocturnal sweats
Seizures
**7.2. How long did you take to seek medical attention after your symptoms appeared?**
Within 24 h
From 24–48 h
Within 1 week
From 1 week - 1 month
From 1–3 months
From 3–6 months
From 6 months - 1 year
More than 1 year
**7.3. In general, since your first symptoms appeared, how long did you take to be seen by an oncologist or hematologist (specialists)?**
Within 24 h
From 24–48 h
Within 1 week
From 1 week - 1 month
From 1–3 months
From 3–6 months
From 6 months - 1 year
More than 1 year
**7.4. How long did it take between the appearance of symptoms and diagnosis of acute lymphoblastic leukemia?**
Within 24 h
From 24–48 h
Within 1 week
From 1 week - 1 month
From 1–3 months
From 3–6 months
From 6 months - 1 year
More than 1 year
**8. Where did you seek your first medical attention?**
Urgency/emergency care (public hospital)
Urgency/emergency care (private hospital)
Primary public care (basic healthcare unit)
Private care medical visit
**8.1. What medical specialist did you consult first?**
**8.2. After your first medical visit, to which medical specialist were you referred?**
**9. How old were you at diagnosis of leukemia? Please, inform months and year.**
**10. Did you have any difficulty during the diagnostic work-up of acute lymphoblastic leukemia? Yes or No**
**10.1. Which were these difficulties?**
Had to visit several medical services before being diagnosed
Had to visit many physicians before being diagnosed
Unavailability of specific/indicated laboratory tests
Difficulty to make an appointment with the referred medical specialist (hematologist or oncologist)
Inconclusive diagnostic work-up
Difficulty in being admitted, then treated in a tertiary hospital
Need to move to another city because treatment was not available in my city
Financial issues
**11. By which healthcare system were you treated?**
Public care
Private care
Clinical trial
Other (specify):
**12. Which was the upfront treatment/protocol indicated by the physician?**
BFM
GMALL
GRAALL
HYPERCVAD
COG
CALGB/C10403
DFCI
GBTLI
Total XV (St. Jude)
Unknown
Chemotherapy (could not be specified)
Chemotherapy with vincristine
Chemotherapy with steroids
Protocol with imatinib or dasatinib (when Ph-positive)
**13. During your first-line treatment, did you receive cranial/brain irradiation? Yes or No**
**14. For how long have you been receiving treatment for acute lymphoblastic leukemia in the first line protocol?**
Up to 3 months
From 3–6 months
From 6–12 months
From 12–18 months
From 18–24 months
From 2 years or more
**15. Did you have any difficulties to start the first line treatment indicated by the physician? Yes or No**
**15.1. Which difficulties did you experience during first line treatment?**
Drug unavailability
Needed to pay to obtain an indicated drug
Had to petition (legal/judicial)
Other (specify):
**16. Did you have to change the upfront treatment schedule? Yes or No**
**16.1. What was the reason to change it?**
Relevant adverse effects
Relapse
Refractory
Moving to another treatment center
Other (specify):
**16.2. Which was the indicated second-line treatment?**
IDA-FLAG
Mitoxantrone plus Ara-C
HYPERCVAD
CLAEG
Nelarabine
Clofarabine
Cytarabine (Ara-C)
GBTLI
Liposomal vincristine
TACL (bortezomib e peg-asparaginase)
REC 17 (St Jude)
BFM
Blinatumomab
Inotuzumab
Unknown
Chemotherapy (could not specify)
Protocol with imatinib/dasatinib
**17. During your treatment, were you informed by your doctor about minimal residual disease (MRD), defined as the presence of residual leukemic cells measured by highly sensitive and specific laboratory tests using bone marrow samples? Yes or No**
**17.1. What MRD status was described by your doctor during your treatment?**
Negative
Positive
Unknown
Inconclusive
**18. Did your doctor indicate bone marrow/stem cell transplantation as a part of your treatment? Yes or No**
**18.1. Did you experience any difficulty to perform bone marrow/stem cell transplantation? Yes or No**
**18.1. Which difficulties did you experience to perform bone marrow/stem cell transplantation?**
Relevant adverse effect
Transplant center unavailability
More than 3 months delay to find a room/bed for transplant
More than 3 months delay to find a matched donor
Other (specify):
**18.2 Where (state and city) did you perform bone marrow transplantation?**
**18.3. Since the physician's indication, how long did you wait to perform bone marrow/stem cell transplantation ?**
Within 1 month
From 1–3 months
From 3–6 months
From 6–12 months
More than 1 year
**18.4. What type of transplantation were you submitted to?**
**Matched related donor (100 % compatibility)**
**Haploidentical related donor (50 % compatibility)**
**Unrelated donor (bone marrow registries)**
**Umbilical cord donor**
**19. Are you satisfied with your treatment? Yes or No**
**19.1. If not, why?**
Adverse effects
Inaccessibility to a multiprofessional healthcare group
Insufficient attention/care of my issues by healthcare professionals
Treatment-related problems that limit my quality of life
Incapacity to work
Incapacity for social life
**20. Have you experienced any of the following health problems due to acute lymphoblastic leukemia treatment?**
Cognitive or memory difficulties/impairment
Mood distress/disorders (like anxiety, depression, panic, post traumatic distress, social displacement/isolation)
Neurologic impairment, such as weakness or numbness in any extremity or part of your body
Diabetes
Systemic arterial hypertension
Blood lipid/cholesterol disorders
Bone fractures
Bone/hip joint disorders, such as osteonecrosis
Myocardial infarction or cerebral/brain stroke
Visual problems (cataracts, glaucoma, visual acuity impairment)
Hearing impairment
Kidney disease
Growth impairment
Liver disease, such as fatty liver, cirrhosis, chronic hepatitis
Lung disease, such obstructive lung disease, emphysema, pneumonitis, fibrosis, Shortness of breath due to lung impairment
Infertility (failure to produce ovules or sperm to have a child)
Heart failure or shortness of breath due to heart disease
Chronic pain (limiting or incapacitating functionality)
Secondary neoplasms
Graft-versus-host disease (GvHD)
Stomach or digestive disorders
Persistent/chronic low platelet count
Hormonal disorders
Mucositis (oral/pharyngeal lesions secondary to chemotherapy)
Thrombosis
Skin disorders
Persistent/chronic low immunity (incapacity to produce antibodies or adequate immunologic responses to attack infections)
Incapacitating/limiting weakness/tiredness
I have not experienced any problem related to ALL treatment
**21. After diagnosis, have you looked for more information about ALL? Yes or No**
**21.1. Where have you searched for this information?**
In hospital specific patient support groups
Physician
Other healthcare professionals: nurse, pharmacists, psychologist, therapist, others
Internet
Blogs or patient web groups
Journals or magazines
Non-governmental organizations (NGOs), patient groups or associations
Other patients
Family members/relatives

Telephone interviews were conducted by a multi-disciplinary team that had received training and was qualified for this task with knowledge about ALL and good communications skills.

If the first attempts were unsuccessful, at least three home or mobile calls were made to contact interviewees within the optimal timeframe. Patient demographics, basic symptoms, diagnosis and treatment data were assessed during the first successful contact. The interviews lasted about 15–20 min. The responses were input into the ABRALE electronic database.

### *Study variables*

The questionnaire (Table 1) consisted of open field and multiple-choice questions, depending on the variables of interest. The basic sociodemographic variables were gender, age at interview, age at ALL diagnosis, presenting symptoms, time to diagnosis, state/city of treatment and residence, relationship with caregiver (when applicable) and healthcare system used (private or public). There were also questions relating to treatment protocols and duration, use of cranial radiotherapy, treatment difficulties and satisfaction, minimal residual disease (MRD) status, allogeneic hematopoietic stem cell transplantation (allo-HSCT) and donor type. From the patient perspective, information regarding difficulties to proceed to an allo-HSCT and treatment related to adverse events were recorded. Secondary variables were dichotomizations or regrouping of recorded primary variables, as required for statistical analysis.

### *Statistical analysis*

The categorical variables are reported as frequencies/proportions. The numeric variables are reported as means, medians, ranges and 95 % confidence intervals (95 % CIs) according to distribution. Potential univariate associations between categorical variables and indication of allo-HSCT or development of adverse events were explored using the chi-square or Fischer exact tests depending on sample distribution. For statistical comparisons, patients were grouped according to the healthcare system accessed (private versus public), while patients that reported access to both health systems (mixed healthcare) were excluded from this comparative analysis. Moreover, patients were grouped and analyzed according to allo-HSCT status, MRD and incidence of adverse effects.

Variables with p-values <0.1 were selected for a multivariate analysis using the logistic regression method.

The Shapiro-Wilk method was applied to test for normality. The Student *t*-test was used to compare parametric variables, while the Wilcoxon-Mann-Whitney (unpaired) test was used to compare non-parametric data. Modified Poisson regression or negative binomial regression models were chosen, according to variable dispersion, to investigate risk factors associated with the cumulative number of adverse events with results being reported as incidence rate ratios (IRRs).^10^

The STATA v.11 and R v.3.5.2 computer programs were used for statistical analysis. The level of significance was set for p-values <0.05

## Results

### *Baseline and diagnostic features*

Four hundred patients were enrolled in this study. Patient characteristics were male 203 (50.7 %) and the mean age 15.7 (range: 0–77) years with 152 (38 %) being over 20 years old. The questionnaires were completed by the patient and not by a caregiver in 128 (32 %) of the cases. Among the remaining 272 cases, the caregiver who responded the interview was one of the patient's parents in 242 (89 %) of the cases. Patients were diagnosed with ALL from 1981 to 2019, while the median date of diagnosis was September 2015 (interquartile range [IQR]: March 2007-February 2018). The median follow-up after ALL diagnosis until this survey was 6.2 years (range: 0.9–38.6).

Most patients 304 (76 %) were from the southeastern or southern regions of Brazil and were treated in the public healthcare system (280 - 70 %), while 79 (20 %) received treatments from private/medical insurance healthcare systems. Forty-one patients (10 %) reported that their treatments were from ‘mixed’ sources, using both the public and private healthcare systems.

**Figure 2:**
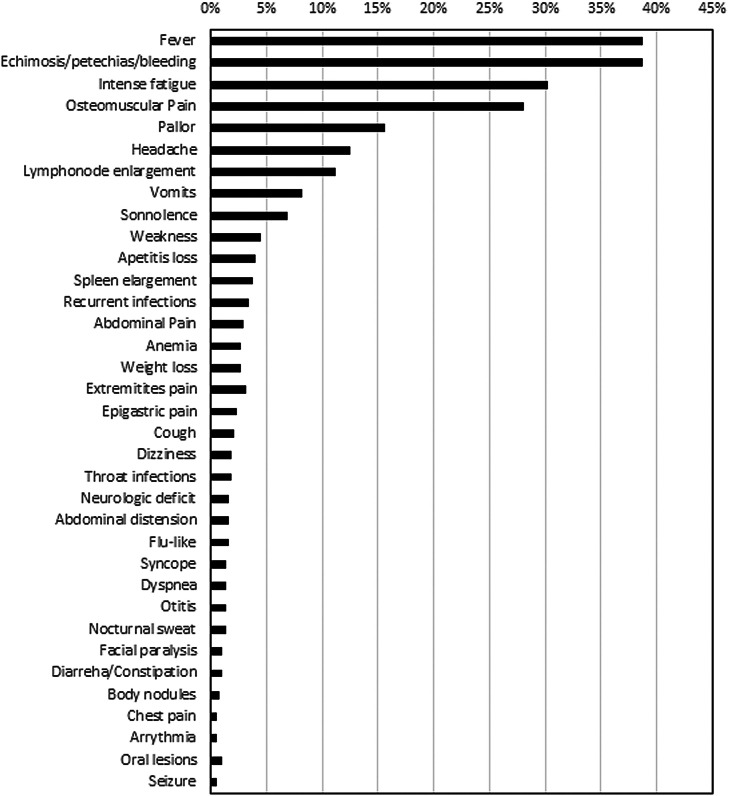
Frequency of symptoms of acute lymphoblastic leukemia patients.

The most commonly reported symptoms at presentation were fever (39 %), bleeding/ecchymosis (38 %), intense fatigue (30 %) and musculoskeletal pain (28 %) (Figure 2). The proportion of patients diagnosed within one week after symptoms onset differed between public (17.9 %) and private healthcare systems (31.1 % - p-value = 0.019). Moreover, diagnostic difficulties were greater for public healthcare patients (35 % versus 22.6 %; p-value = 0.034). The main diagnostic problems reported by patients were referrals to multiple institutions (65/134 - 48.7 %), repeated medical visits (80/134 - 59.8 %), inconclusive diagnostic test results (35/134 - 26 %), inaccessibility to essential laboratory tests (17/134 - 13 %) and difficulty to be admitted in a tertiary hospital (4/134 - 3 %).

### *Treatment and MRD: patients’ understanding and experiences*

All but two patients were treated from 1992 to January 2019 (one in 1981 and one in 1986) with a median follow-up from diagnosis to study survey date of 6.2 years (range: 0.9–38.6). The great majority of patients (91 %; 364/400) did not know their treatment protocol. Thus, only 36 (9 %) patients knew their treatment protocol; overall, patients were able to mention eight protocols (Table 2); two were reported most, the GBTLI (10 - 27.8 %) and the Berlin-Frankfurt-Münster (BFM in 8 - 22.2 %) protocols.

**Table 2:** Distribution of patients who reported ALL treatment protocols.

Treatment protocol	n (%)
Brazilian Childhood Cooperative Group for Treatment of ALL in children (GBTLI)^3^^,^^13^	10 (27.8)
Berlin-Frankfurt-Münster (BFM)^11^	8 (22.2)
Children's Oncology Group (COG) 28, 31/ C10403^32^	2 (5.6)
St. Jude's Hospital TOTALXV^27^	2 (5.6)
Group for Research on Adult ALL (GRAALL)^26^	2 (5.6)
Dana Farber Cancer Institute (DFCI)^25^	1 (2.8)
Hyper-CVAD^23^	1 (2.8)
German Multicenter ALL (GMALL)^24^	1 (2.8)
Tyrosine kinase inhibitor (TKI)-based protocol (imatinib or dasatinib)	4 (11.1)
Chemotherapy containing vincristine and/or steroids	5 (13.8)

In general, 70 patients (17.5 %) required modifications of treatment attributed to the following reported causes: 32 (45.7 %) upfront refractoriness to chemotherapy, 26 (37.1 %) severe adverse events and three (4.3 %) relapses. Four patients (5.7 %) modified their treatment because they moved to another treatment center and five (7.1 %) did not know why there was a modification.

Forty-eight patients (12 %) had at least one treatment difficulty: 26 (54.1 %) reported that a necessary drug was not available at the time, 12 (25 %) reported the need to buy medications themselves and ten (20.8 %) had to petition to demand drugs to start their indicated treatment. Minimal residual disease (MRD) status was remembered by 99 (24 %) of the patients; it was positive in 22 (23.9 %) cases.

More than half of patients 206 (51.5 %) reported a treatment duration of two years or more, while the treatment lasted six months or less for 87 (21.7 %); among these patients 27.6 % (24/87) were submitted in first remission to allo-HSCT, justifying the shortened treatment protocol.

Cranial radiotherapy was reported by 109 patients (27.2 %) as part of their treatments. Interestingly, comparing the group of patients reporting cranial irradiation versus those treated without cranial irradiation, univariate analysis showed that a higher frequency of cranial irradiation was associated with increased age (>15 years old: 35.0 % versus 19.1 %; p-value = 0.005) and indication of allo-HSCT (51.7 % versus 20.1 %; p-value <0.001). While being diagnosed after September 2015 was associated with less irradiation (21.0 % versus 33.1 %; p-value = 0.01). In multivariate analysis, indication of allo-HSCT remained associated with a higher risk (odds ratio [OR]: 4.60; 95 %CI [95 % confidence interval]: 2.69–7.79; p-value <0.001) of cranial irradiation. In contrast, being diagnosed after September 2015 was associated with a lower risk (OR: 0.57; 95 % CI: 0.35–0.92; p-value = 0.021) of cranial irradiation and age was not significant in this multivariate analysis

### Indication of allogeneic hematopoietic stem cell transplantation and challenges

In this study, 91 patients (22.7 %) reported referral for allo-HSCT by their physician. At the time of the study survey, 64 (70.3 %) had already undergone allo-HSCT. Among these patients, 31 (48 %) had received grafts from matched related donors, 24 (38 %) from matched unrelated donors, six (9 %) from haploidentical donors and three (5 %) had receiving umbilical cord grafts. Among the patients not submitted to transplants, 19 (20.9 %) were still waiting for donors for the procedure and eight (8.8 %) were waiting for hospital admission.

The transplantation centers were located mainly in the southeastern and southern regions of Brazil. Most patients 34 (53.1 %) needed to move to another city to proceed with the allo-HSCT, while 16 (25.0 %) needed to move far beyond, from one state to another, and 30 (46.9 %) underwent allo-HSCT in the same city as they were living. Forty patients (62 %) reported that more than three months elapsed between the indication for allo-HSCT and the transplant procedure; in 27 (42 %) the waiting time was longer than six months. Seventeen patients (27 %) reported difficulties with the allo-HSCT procedure: for 11 (65 %), the delay was more than three months to find a donor and arrange donor-related procedures, and two patients reported a long delay for hospital admission due to bed/room unavailability. No significant difference was found on analyzing the transplant delay of more than three months in respect to the donor type: matched related 13/31 (42 %) versus matched unrelated (7/24; 29 %; p-value = 0.24).

The following factors were associated with a higher probability of indications for allo-HSCT: MRD positivity (OR: 3.22; 95 %CI: 1.07–9.66; p-value = 0.037), cranial radiotherapy (OR: 4.36; 95 %CI: 1.61–11.8; p-value = 0.004) and age (OR: 1.03/year; 95 %CI: 0.99–1.06; p-value = 0.068).

### *Patient-reported outcomes*

Overall, 390 (97.5 %) patients reported satisfaction with their treatments. The remaining 10 (2.5 %) patients were unsatisfied due the following reasons: long-term adverse events, insufficient attention to their issues by healthcare professionals or inability to resume social and working relationships.

**Figure 3:**
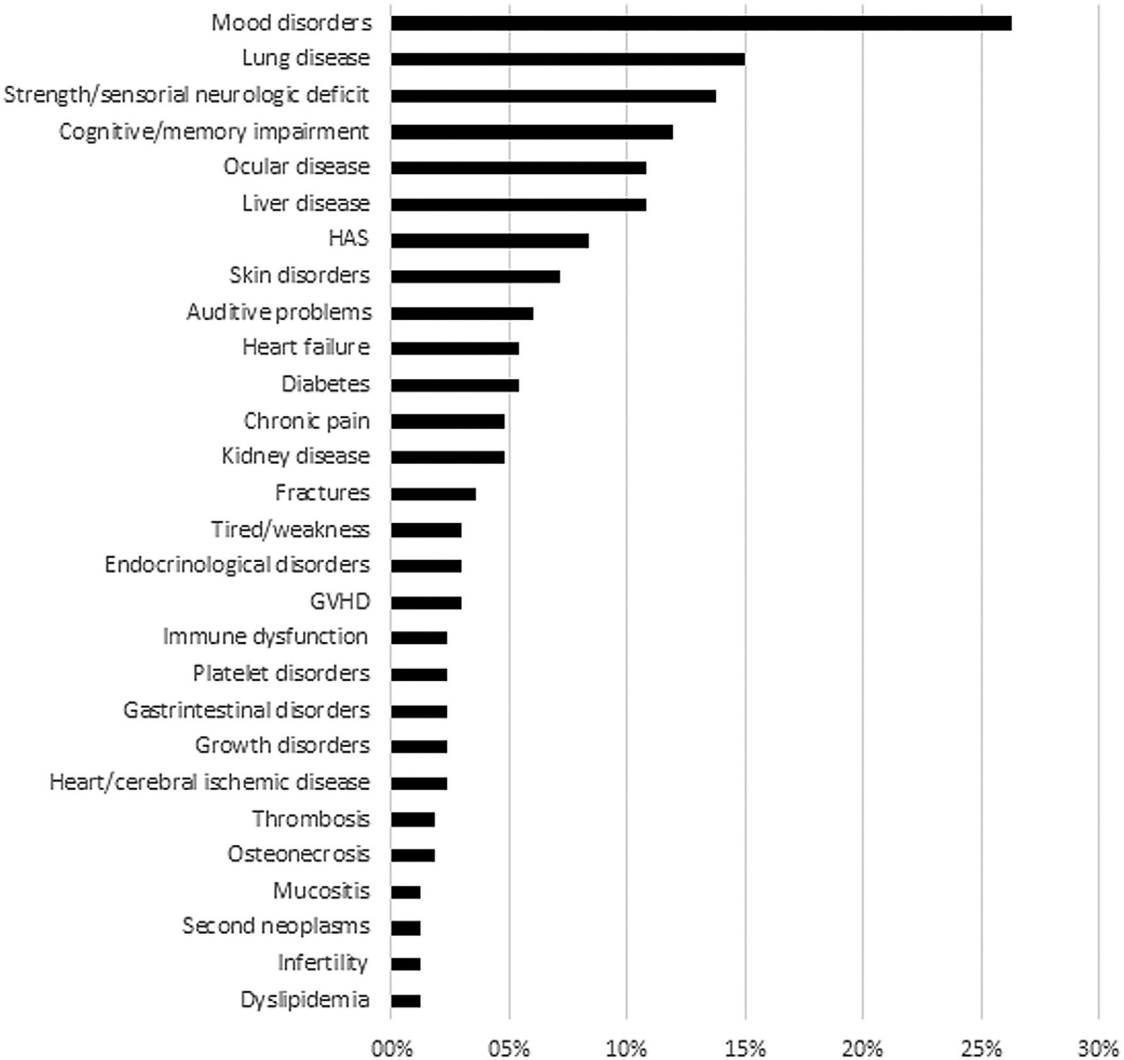
Frequency of adverse events reported by acute lymphoblastic leukemia patients. HAS: Systemic arterial hypertension; GVHD: Graft-versus-host disease.

Despite the high satisfaction rate, 167 (41.7 %) reported at least one ongoing treatment-related adverse event. The most frequently reported adverse events were mood disorders (44/167 - 26.3 %), neurologic impairment characterized by sensorial/strength deficiencies (23 - 13.8 %) or cognitive/memory disorders (20 - 12.0 %), visual problems (18 - 10.8 %), including cataracts, glaucoma and low visual acuity, lung disease (25 - 15 %), such as chronic obstructive/restrictive diseases and emphysema, and liver disease (18 - 10.8 %), such as fatty liver, chronic hepatitis and cirrhosis. The reported frequencies of clinically-relevant adverse events are listed in (Figure 3).

The risk of experiencing at least one adverse event was associated with: age (OR: 1.019/year; 95 %CI: 1.00–1.03; p-value = 0.003), female gender (OR: 1.52; 95 %CI: 0.99–2.31; p-value = 0.05), indication for allo-HSCT (OR: 1.67; 95 %CI: 1.004–2.78; p-value = 0.048) and living in a large city or state capital versus smaller cities (OR: 1.92; 95 %CI: 1.26–2.92; p-value = 0.002). Furthermore, the median and mean cumulative adverse event rates were 1 and 1.58 (range: 1–6 per patient). This analysis identified that indication for allo-HSCT (IRR: 1.23; 95 %CI: 1.03–1.49; p-value = 0.03) and age (IRR: 1.004/year; 95 %CI: 1.000–1.007; p-value = 0.04) were variables associated with increased relative incidence ratios of cumulative adverse events. Table 3 summarizes most relevant differences between patients indicated for allo-HSCT and those without indication.

**Table 3:** Univariate analysis comparing patient age, central nervous system irradiation and adverse events in respect to patients who underwent allogeneic hematopoietic stem cell transplantation (allo-HSCT).

Variable	allo-HSCT (*n* = 91)	No allo-HSCT (*n* = 309)	p-value
Age - mean (95 % CI)	22.8 (19.4–26.3)	13.6 (11.7–15.5)	<0.001
Follow-up - median (IQR)	4.7 (6.4)	3.9 (5.6)	0.13
CNS irradiation - n (%)	47 (51.6 %)	62 (20.1 %)	<0.001
MRD positivity - n/total (%)	11/17 (39.3 %)	11/64 (17.1 %)	0.033
Ophthalmologic disease - n (%)	14 (15.4 %)	4 (1.3 %)	<0.001
Neurologic dysfunction - n (%)	9 (9.9 %)	14 (4.5 %)	0.053
Lung disease - n (%)	12 (13.2 %)	13 (4.2 %)	0.005
Liver disease - n (%)	8 (8.8 %)	10 (3.2 %)	0.039
Cumulative adverse events	IRR*: 1.23; (95 % CI: 1.03–1.49)		0.03
Memory/cognitive impairment - n (%)	6 (6.6 %)	14 (4.5 %)	0.291
Mood disorders - n (%)	9 (9.9 %)	35 (11.3 %)	0.7

CI: confidence interval; IQR: interquartile range; *IRR: incidence rate ratio calculated by negative binomial regression model; MRD: minimal residual disease; CNS: central nervous system.

## Discussion

This study provides interesting findings about the frequency of adverse effects of ALL patients in Brazil, a large middle-income country with high socioeconomic disparities and an underfunded public healthcare system. Unexpectedly, this study found that 21.7 % of ALL patients were treated with short protocols, reporting durations of less than six months,^3^ as most ALL protocols last more than two years.^3^^,^^11^

In contrast, 27.2 % of patients reported treatments including cranial irradiation, which might reflect higher than expected rates of irradiation, as this approach should be reserved for patients with central nervous system (CNS) infiltration (CNS3 status), a condition that affects about 10 % of ALL patients.^12^ Furthermore, in current ALL protocols the indication of cranial irradiation is being replaced by intensifying intrathecal chemotherapy and CNS penetrating therapies.^13^ Interestingly, in this study, many patients received cranial irradiation as part of the allo-HSCT treatment (51.7 %), probably during the conditioning regimen with the rate of CNS irradiation decreasing from 33.1 % to 21.0 % over time, as noted by comparing reports of patients treated before and after September 2015. In addition, in the subset of patients treated more recently and without an allo-HSCT, the reported percentage of CNS irradiation was 13.7 %, which is approaching the expected rate described in the literature,^12^ demonstrating that patient care is changing due to current protocols. However, one must interpret these findings cautiously, as they may be biased by the patients’ misinterpretation of the questionnaire and memory.

This study revealed a variety of more than eight protocols reported by a small subset of 36 patients and observed that increasing age is associated with the risk of adverse events. There is strong evidence that the knowledge, experience and specific adjustments of each center according to the available facilities are critical to achieve favorable outcomes in ALL.^1^^,^^2^, ^3^, ^4^^,^^11^^,^^12^^,^^14^ Moreover, experience in the development of pediatric ALL protocols in Brazil (1980–2009) provided a remarkable improvement in the five-year overall survival rates (from 34.1 to 79.3 %).^14^ We believe that treatment disparities may be negatively affecting the outcomes of young adult patients (20–40 year olds), representing 21 % (84/400) of this study cohort who, in the Brazilian reality, are usually treated without standardized protocols.

In accordance to the literature,^5^^,^^6^^,^^13^ mood disorders and cognitive/memory impairment were frequently reported adverse events. Interestingly, two studies observed that mood disorders may also affect parents and caregivers.^15^^,^^16^ In this study, almost 60 % of the interviewed population were caregivers/parents, who therefore could unconscientiously influence the mood disorder reports. Patient fitness may also play a role in the development of adverse events,^16^ an issue that may be suitable to multi-professional interventions.^15^^,^^16^

A surprising new finding was that 15 % of the reported adverse events were lung problems, such as chronic obstructive/restrictive diseases. Despite the restricted use of classically pneumotoxic drugs in ALL protocols, some studies have reported deteriorations in lung function in ALL survivors,^17^^,^^18^ especially related to oxygen diffusion capacity, although the exact reasons for this lung dysfunction have not been clarified yet. In fact, they are not frequently reported in clinical studies,^19^^,^^20^ except for in allo-HSCT series,^17^^,^^18^ and are mainly attributed to total body irradiation conditioning and chronic graft-versus-host disease.^17^, ^18^, ^19^ Two hypotheses may explain the reported lung dysfunction. First, it could be related to patient's misinterpretation, leading to confusion between typical tobacco-induced chronic obstructive lung disease and post-treatment induced lung dysfunction. A second hypothesis would be that long-term methotrexate exposure during maintenance therapy and recurrent lung infections might affect lung function. Some studies reported significant rates of dyspnea among these patients^6^^,^^7^ and there is some evidence that recurrent lung infections, especially cytomegalovirus infections, and allo-HSCT are risk factors for chronic pulmonary disease among ALL survivors.^6^^,^^7^^,^^17^^,^^18^

A French study reported an association of MRD positivity with the risk of adverse events and quality of life of patients with ALL,^7^ a finding that could be related to exposure to more intensive treatment arms and a higher rate of allo-HSCT among patients. The limited number of patients (*n* = 99) that could recall MRD information and its reliability, when obtained from patients, prevents further analysis in this study.

The incidence of symptomatic clinically relevant adverse events, such as thrombosis and bone problems range from 6 to 13 % and 4.0–6.8 %, respectively in clinical series.^19^, ^20^, ^21^ The present study observed a relatively lower reported rate of thrombosis (1.8 %) and a similar rate of bone problems (5.4 %), mainly bone fractures and osteonecrosis(Figure 3). Differences between clinical series and patient-reported outcomes should be interpreted cautiously because patient-reported outcomes might be prone to describe later events and psychosocial adverse events, while clinical series trend to report more short- and medium-term effects from the physicians’ viewpoint. This highlights the reason why patient-reported outcomes could be so important in complementing our knowledge about patient care and how health professionals should concentrate their efforts to improve care.

In agreement with the literature, this study found that indication of allo-HSCT was associated with age, MRD positivity and CNS irradiation in this cohort mainly represented by pediatric, adolescent and young adult patients, as these factors are usually high-risk features.^22^ However, recall bias, questionnaire misinterpretation and selection bias should be considered as limitations for arriving at conclusive interpretations in this study. Furthermore, CNS infiltration, especially CNS2 status, by itself is not being considered a strong high-risk criterion to guide transplant indication in current protocols.^22^

Unexpectedly, this study found that approximately 20 % of patients experienced delays of more than three months for allo-HSCT due to the lack of a donor or hospital bed unavailability, issues that might be improved by administrative interventions.

Generally, most (70 %) patients experienced a diagnostic delay, which was defined as an elapsed time of more than one week from symptoms onset until diagnosis, whereas 27 % were diagnosed beyond a four-week threshold. Furthermore, we also observed significantly different frequencies of patients within the one-week target between private (31.1 %) and public (17.9 %) healthcare. Secondly, this finding was also reinforced by a higher proportion of diagnostic difficulties in public (35.0 %) compared to private (22.6 %) care. These findings are probably reflecting a suboptimal and heterogeneous access to healthcare facilities.

On the other hand, there is no established optimal timing for ALL diagnosis. One review addressing delays in childhood cancers, reported that the median diagnostic delay in acute leukemia patients ranges from 3 to 5.4 weeks,^29^ whereas one Canadian study reported a median time of eight days from symptoms onset to specialty evaluation; indeed 11 days from symptoms onset to initiate treatment for pediatric patients with acute leukemia.^30^ In this context, we chose a one-week threshold to evaluate this outcome because many of these patients presented fever associated with neutropenia, thus justifying a more urgent approach.

The presenting clinical picture of ALL may vary; sometimes it presents as musculoskeletal symptoms, including arthritis, arthralgia and bone pain, confounding it with rheumatologic disorders. This might be a really challenging scenario for physicians and also for patients to recognize their own symptoms,^31^^,^^32^ leading to the need of an extensive diagnostic work-up, which contributes to diagnostic delay.^31^^,^^32^ Physicians must be aware of this condition, with efforts to avoid steroids, promptly requesting bone marrow aspirations and closely following up these patients, as many laboratory and imaging test abnormalities might appear later on.^31^^,^^32^ Compared to rheumatologic disorders, the involvement of hips, knees, presence of unexplained anemia or thrombocytopenia, elevated uric acid or lactate dehydrogenase increase the probability of a diagnosis of ALL.^31^^,^^32^

## Conclusions

This study revealed very important findings related to diagnosis, treatment and adverse events of Brazilian ALL survivors. Diagnostic delays are a very important issue to be faced. Study limitations, such as recall bias, the lack of standardized treatment protocols, especially for young adults, might lead to a 20–30 % rate of unoptimized treatments. Estimated frequencies in the delay of allo-HSCT of 62 % and for adverse events of 41.7 % were observed, the latter mainly represented by mood disorders, neurologic impairment and lung disease. Further efforts are needed to standardize ALL care, shorten delays for allo-HSCT and improve patient care focusing on neuropsychological and pulmonary issues.

## Funding

AMIGOH - Diretoria de Responsabilidade Social - Hospital Israelita Albert Einstein

## Data availability statement

The data that support these findings are available from the corresponding author upon reasonable request.

## Conflicts of interest

The authors declare no conflict of interest.
